# Garlic supplementation for the treatment of chronic liver disease: a meta-analysis of randomized controlled trials

**DOI:** 10.4314/ahs.v23i2.47

**Published:** 2023-06

**Authors:** Liu Xiaohui, Li Jinqi, Xie Xiaofang, Shi Zhiqiang, Niu Renxiu

**Affiliations:** 1 College of traditional Chinese medicine, Inner Mongolia Medical University, Hohhot, 010110, China; 2 Department of Pharmacy, Sichuan Academy of Medical Science & Sichuan Provincial People's Hospital, School of Medicine, University of Electronic Science and Technology of China, 610072 Chengdu, China; 3 Department of Pharmacy, Sichuan Academy of Medical Science & Sichuan Provincial People's Hospital, School of Medicine, University of Electronic Science and Technology of China, 610072 Chengdu, China

**Keywords:** Garlic, chronic liver disease, randomized controlled trials, meta-analysis

## Abstract

**Introduction:**

The efficacy of garlic supplementation for chronic liver disease remains controversial. We conduct a meta-analysis to explore the influence of garlic supplementation versus placebo on the treatment of chronic liver disease.

**Methods:**

We have searched PubMed, EMbase, Web of science, EBSCO, and Cochrane library databases through September 2021 for randomized controlled trials (RCTs) assessing the efficacy of garlic supplementation versus placebo for chronic liver disease. This meta-analysis is performed using the random-effect model.

**Results:**

Four RCTs and 212 patients are included in the meta-analysis. Overall, compared with control group for chronic liver disease, garlic supplementation is associated with significantly reduced alanine aminotransferase (ALT), aspartate-aminotransferase (AST), total cholesterol, low density lipoprotein (LDL) and weight, but demonstrates no substantial impact on the incidence of adverse events.

**Conclusions:**

Garlic supplementation is effective to treat chronic liver disease.

## Introduction

Chronic liver diseases such as non-alcoholic fatty liver disease (NAFLD) widely occur in adults 1-4. They result in a broad spectrum of hepatic damage from simple hepatic steatosis to steatohepatitis, which can progress to fibrosis or even carcinoma 5-7. Chronic liver diseases may also increase the risk of cardiovascular events 8. Progressive cirrhosis also significantly increases the requirement of liver transplantation 9.

Garlic, one kind of vegetable, has been used as a medicinal plant in complementary medicine in many countries. It has a broad spectrum of organosulfur compounds, such as alliin, allicin, vinyl dithiins and ajoene, which have antioxidant and anti-inflammatory properties 10, 11. Some trials reported the therapeutic potential of garlic and its derivatives against cardiovascular diseases and cancers 12, 13. Emerging evidence suggested that garlic supplementation exerted protective roles in the management of NAFLD by regulating inflammation, hepatic lipid metabolism and modulating gut bacteria flora 14-16.

However, the benefit of garlic supplementation for chronic liver disease has not been well established. Recently, several studies on the topic have been published, and the results have been conflicting 17-19/span>]. With accumulating evidence, we therefore perform this meta-analysis of RCTs to explore the efficacy of garlic supplementation versus placebo for chronic liver disease.

## Materials and methods

Ethical approval and patient consent are not required because this is a systematic review and meta-analysis of previously published studies. The systematic review and meta-analysis are conducted and reported in adherence to PRISMA (Preferred Reporting Items for Systematic Reviews and Meta-Analyses) 20, 21.

### Search strategy and study selection

Two investigators have independently searched the following databases (inception to September 2021): PubMed, EMbase, Web of science, EBSCO and Cochrane library databases. The electronic search strategy is conducted using the following keywords: “liver disease” OR “steatohepatitis” AND “garlic”. We also check the reference lists of the screened full-text studies to identify other potentially eligible trials.

The inclusive selection criteria are as follows: (i) patients are diagnosed with chronic liver disease; (ii) intervention treatments are garlic supplementation versus placebo; (iii) study design is RCT.

### Data extraction and outcome measures

We have extracted the following information: author, number of patients, age, female, weight, body mass index and detail methods in each group etc. Data have been extracted independently by two investigators, and discrepancies are resolved by consensus. We also contact the corresponding author to obtain the data when necessary. The primary outcomes are ALT and AST. Secondary outcomes include total cholesterol, LDL, weight change and adverse events.

### Quality assessment in individual studies

Methodological quality of the included studies is independently evaluated using the modified Jadad scale 22. There are three items for Jadad scale: randomization (0-2 points), blinding (0-2 points), dropouts and withdrawals (0-1 points). The score of Jadad Scale varies from 0 to 5 points. An article with Jadad score≤2 is considered to have low quality. If the Jadad score≥3, the study is thought to be of high quality 23, 24.

### Statistical analysis

We estimate the standard mean difference (SMD) or mean difference (MD) with 95% confidence interval (CI) for continuous outcomes and odd ratio (OR) with 95%CI for dichotomous outcomes. A random-effects model is used regardless of heterogeneity. Heterogeneity is reported using the I2 statistic, and I2 > 50% indicates significant heterogeneity 25. Whenever significant heterogeneity is present, we search for potential sources of heterogeneity via omitting one study in turn for the meta-analysis or performing subgroup analysis. All statistical analyses are performed using Review Manager Version 5.3 (The Cochrane Collaboration, Software Update, Oxford, UK).

## Results

### Literature search, study characteristics and quality assessment

**Figure 1:**
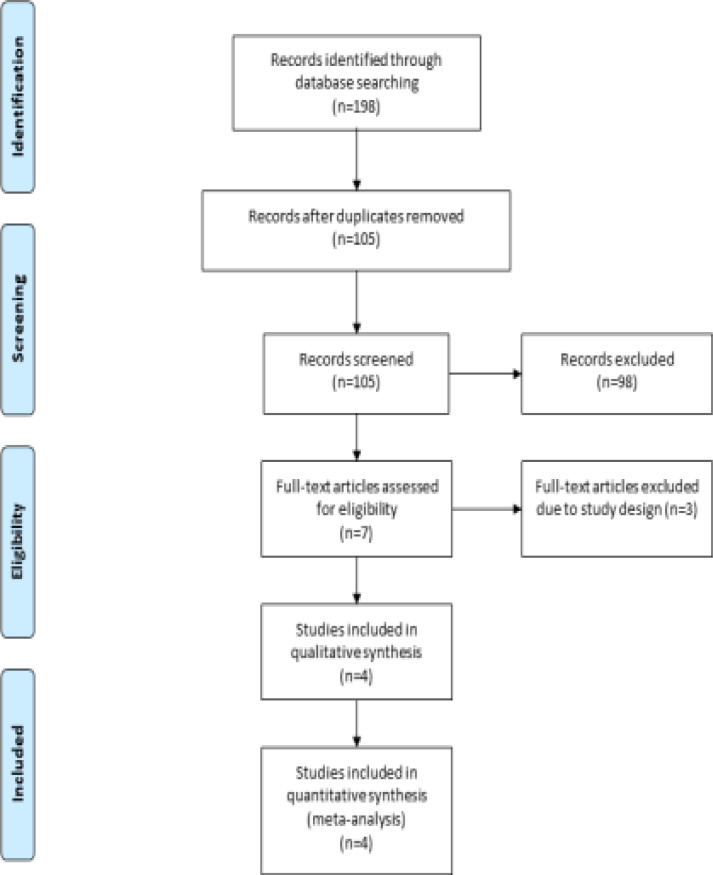
Flow diagram of study searching and selection process

A detailed flowchart of the search and selection results is shown in Figure 1. 198 potentially relevant articles are identified initially. Finally, four RCTs are included in the meta-analysis 17-19, 26. The baseline characteristics of four eligible RCTs in the meta-analysis are summarized in Table 1. The four studies are published between 2014 and 2021, and total sample size is 416. The doses of garlic supplementation are different in each RCT. Among the four studies included here, three studies report ALT , AST 17-19, two studies report total cholesterol, LDL and weight 17, 18 and two studies report adverse events 19, 26. Jadad scores of the four included studies vary from 4 to 5, and all four studies are considered to have high quality according to quality assessment.

**Table 1:** Characteristics of included studies

NO.	Author	Garlic group						Control group						Jada scores
		Number	Age (years)	Female (n)	Weight (kg)	Body mass index (kg/m2)	Methods	Number	Age (years)	Female (n)	Weight (kg)	Body mass index (kg/m2)	Methods	
1	Soleimani 2020	55	45.6±11.3	30	82.6±14.3	30.7±5.2	800 mg garlic daily for 15 weeks	55	42.9±12.21	34	79.8±14.7	28.6±6.4	placebo	4
2	Sangouni 2020	45	45.2±12.4	12	89.8±11.9	30.2±3.1	four tablets of garlic daily (each tablet contained 400 mg garlic powder) for 12 weeks	43	44.2±11.1	19	90±13.6	31.6±3.8	placebo	5
3	Kim 2017	36	54.5±12.2	11	70.9±13.3	-	two sachets garlic/day for 12 weeks	39	54.2 ± 9.3	9	72.2±12.6	-	placebo	4
4	Kim 2014	100	44 (20-79), median (range)	22	-	-	480 mg pennel for 12 weeks	43	44 (24-77)	16	90±13.6	31.6±3.8	placebo	4

### Primary outcomes: ALT and AST

**Figure 2:**
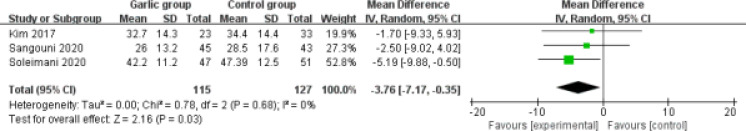
Forest plot for the meta-analysis of ALT

**Figure 3:**
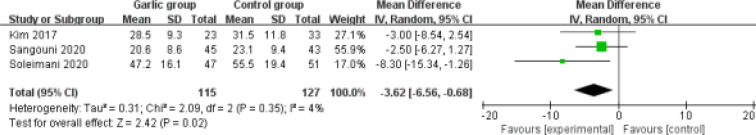
Forest plot for the meta-analysis of AST

These outcome data are analysed with the random-effects model, and compared to control group for chronic liver disease, garlic supplementation is associated with significantly reduced ALT (MD=-3.76; 95% CI=-7.17 to -0.35; P=0.03) with no heterogeneity among the studies (I2=0%, heterogeneity P=0.68, Figure 2) and AST (MD=-3.62; 95% CI=-6.56 to -0.68; P=0.02) with low heterogeneity among the studies (I2=4%, heterogeneity P=0.35, Figure 3).

### Sensitivity analysis

Low heterogeneity is observed among the included studies for the primary outcomes, and thus we do not perform sensitivity analysis via omitting one study in turn to detect heterogeneity.

### Secondary outcomes

**Figure 4:**
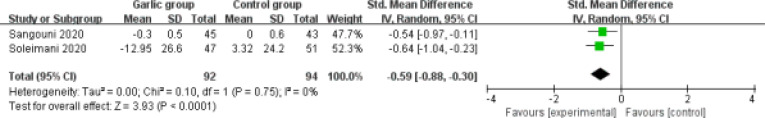
Forest plot for the meta-analysis of total cholesterol

**Figure 5:**
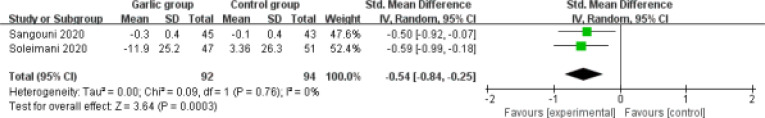
Forest plot for the meta-analysis of LDL

**Figure 6:**
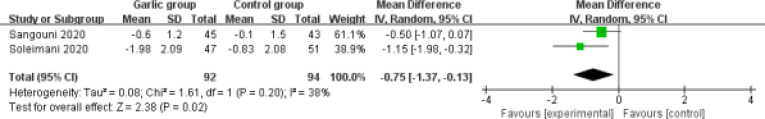
Forest plot for the meta-analysis of weight

**Figure 7:**
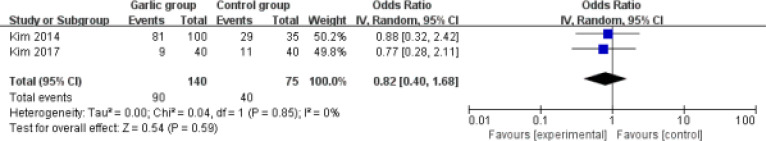
Forest plot for the meta-analysis of adverse events

In comparison with control group for chronic liver disease, garlic supplementation can substantially reduce total cholesterol (SMD=-0.59; 95% CI=-0.88 to -0.30; P<0.0001; Figure 4), LDL (SMD=-0.54; 95% CI=-0.84 to -0.25; P=0.0003; Figure 5) and weight (SMD=-0.75; 95% CI=-1.37 to -0.13; P=0.02; Figure 6), but shows no substantial impact on the incidence of adverse events (OR=0.82; 95% CI=0.40 to 1.68; P=0.59; Figure 7).

## Discussion

NAFLD serves as the leading cause of chronic liver disease [27, 28]. Type 2 diabetes mellitus is the independent risk factor of cirrhosis, hepatocellular carcinoma and double the death risk from liver cirrhosis 29. Liver fat accumulation may result in simple triglyceride accumulation (steatosis), non-alcoholic steatohepatitis, cirrhosis, and even hepatocellular carcinoma 30. However, current pharmacologic agents are not effective for the treatment of these chronic liver disease. Insulin resistance and inflammation was confirmed to have critical roles in the progression of chronic liver disease 31.

Our meta-analysis included four eligible RCTs and 416 patients, and the results confirmed that garlic supplementation exerted important beneficial effect on hepatic function for chronic liver diseases, as evidenced by the reduced ALT, AST, total cholesterol, LDL and weight. Garlic and its derivatives can mitigate the hepatic steatosis by down-regulating the expression of sterol regulatory element-binding protein-1c (SREBP1c) and up-regulating the expression of peroxisome proliferator-activated receptor α (PPAR α ) and carnitine palmitoyltransferase-1 (CPT-1) 32.

Regarding the sensitivity analysis, although there is no significant heterogeneity, several factors may produce some bias. Firstly, chronic liver diseases included NAFLD, alcohol and virus-induced liver diseases, which were caused by different etiologies. Secondly, the doses and forms of garlic supplementation varied in each RCTs, detail in Table 1. Thirdly, the severity levels of chronic liver diseases were various, and may affect the efficacy assessment of garlic supplementation. In terms of safety, adverse events were not increased after garlic supplementation in our meta-analysis, and these adverse events were generally mild and acceptable.

Our meta-analysis also has some important limitations. Firstly, our analysis is based on four RCTs, and two of them have a relatively small sample size (n<100). Overestimation of the treatment effect is more likely in smaller trials compared with larger samples. Although there is no significant heterogeneity, different doses and forms of garlic supplementation may produce some bias. Finally, different etiologies and severity levels of chronic liver diseases may affect the efficacy assessment of garlic supplementation.

## Conclusions

Garlic supplementation provides additional benefits to treat chronic liver disease.
